# Ruptured hepatic metastases of cutaneous melanoma during treatment with vemurafenib: an autopsy case report

**DOI:** 10.1186/s12907-015-0015-3

**Published:** 2015-09-03

**Authors:** Takuto Nosaka, Katsushi Hiramatsu, Tomoyuki Nemoto, Yasushi Saito, Yoshihiko Ozaki, Kazuto Takahashi, Tatsushi Naito, Kazuya Ofuji, Hidetaka Matsuda, Masahiro Ohtani, Hiroyuki Suto, Yoshiaki Imamura, Yasunari Nakamoto

**Affiliations:** Second Department of Internal Medicine, Faculty of Medical Sciences, University of Fukui, Fukui, Japan; Department of Pathology, University of Fukui Hospital, Fukui, Japan

## Abstract

**Background:**

The spontaneous rupture of hepatic metastases is rare compared to that of primary hepatic tumors. In addition, vemurafenib, a selective inhibitor of the mutant BRAF protein or gene product, has been reported to be extremely effective in patients with metastatic melanoma who harbor a *BRAF V600E* mutation.

**Case presentation:**

A 44-year-old female had previously undergone surgery for resection of a malignant melanoma in the lower right leg. Four years later, hepatic metastases became apparent, and transcatheter arterial embolization (TAE) was performed. Then she underwent treatment with vemurafenib. The size of the hepatic metastases markedly decreased. Two months later, they enlarged rapidly and ruptured, requiring emergency TAE. However, the patient developed hemorrhagic shock and died of renewed intra-abdominal bleeding on the 26th postoperative day.

**Conclusions:**

This is a rare case of ruptured hepatic metastases of malignant melanoma during treatment with vemurafenib. Postmortem examination and immunohistochemical analysis indicated reactivation of the mitogen-activated protein kinase pathway in the metastatic tumor, suggesting secondary resistance to vemurafenib as the possible underlying mechanism.

## Background

Hepatic metastases are observed in 68 % of all patients with malignant melanoma at autopsy [1]. However, few cases of ruptured hepatic metastases of melanoma have been reported [2–9]. Recently, it has been reported that treatment with vemurafenib results in complete or partial tumor regression in >80 % of melanoma patients with the *BRAF V600E* mutation [10]. The following case report describes a rare complication of ruptured hepatic melanoma that occurred during treatment with vemurafenib. The postmortem examination of the present case may provide insight into the mechanism underlying this tumor’s secondary resistance to vemurafenib and the subsequently fatal rupture.

## Case presentation

**Fig. 1:**
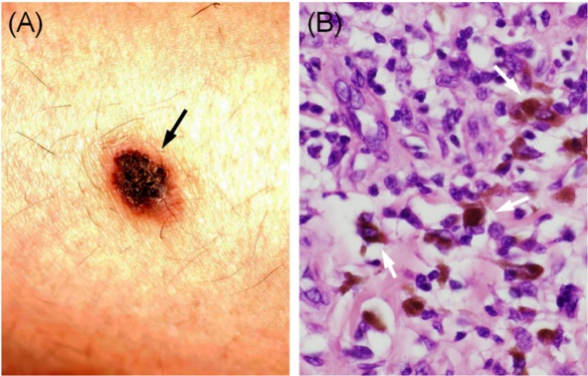
Gross examination and histologic finding of the cutaneous melanoma. The pigmented skin is located on the lower right leg (*black arrow*) (**a**). The resected specimen contained granular, brown, cytoplasmic pigmented cells (*white arrow*) (hematoxylin-eosin stain, ×400) (**b**)

In August 2008, a 44-year-old female had undergone surgery for resection of a malignant melanoma in the right lower leg and a right inguinal metastatic lymph node (Fig. 1), followed by chemotherapy with doxorubicin, adriamycin, vincristine, and interferon beta (DAV-feron). In March 2012, computed tomography (CT) revealed brain and lung metastases, so the patient began radiation therapy to treat these lesions.

In September 2012, the patient was admitted to our hospital for back pain. Abdominal CT and magnetic resonance imaging detected new multiple hepatic metastases of melanoma. A transcatheter arterial infusion of cisplatin was administered, and transcatheter arterial embolization (TAE) was performed. In October 2012, she began treatment with vemurafenib, based on the finding of a positive *BRAF V600E* mutation in the resected primary site of the skin, which was analyzed by direct sequencing analysis using DNA from the paraffin-embedded primary cutaneous melanoma. She tolerated the treatment remarkably well, and the size of the multiple hepatic and lung metastases decreased, while the size of the brain metastases did not. In addition, the serum concentration of 5-S-cysteinyldopa (5-S-CD), a biological marker of melanoma progression, was also decreased from 40.1 ng/mL to 5.2 ng/mL.

**Fig. 2:**
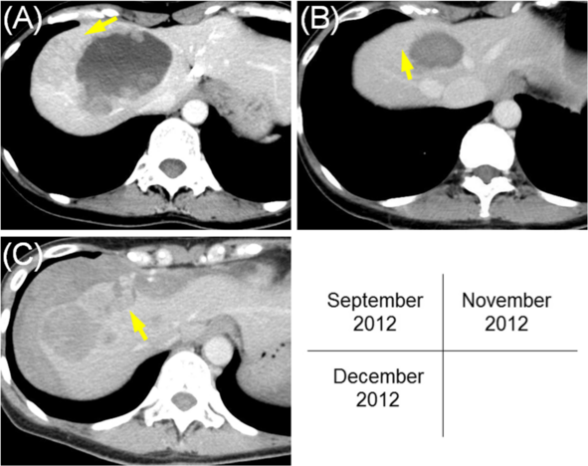
Sequential images of abdominal computed tomography (CT). These images are from September 2012 (**a**), November 2012 (**b**), and December 2012 (**c**). There is a metastatic tumor in the right lobe of the liver (**a**). Initially, the hepatic metastases considerably respond to vemurafenib treatment, and they become almost invisible (**b**). Later, they grow rapidly and rupture, resulting in a large amount of free fluid within the peritoneal cavity surrounding the liver (**c**). The *yellow arrow* demonstrates the same tumor in each image

In December 2012, she suddenly developed severe abdominal pain. Abdominal CT revealed ruptured hepatic metastases accompanied by massive intra-peritoneal hemorrhage. A retrospective and sequential analysis of the CT images suggested that a part of the liver metastases had enlarged rapidly and then ruptured with intratumoral hemorrhage during vemurafenib treatment (Fig. 2). An emergency TAE was performed by selective occlusion of the right hepatic artery using gelatin sponge particles. The postoperative course was uneventful for several days. However, on the 26th postoperative day, she developed hemorrhagic shock and died of renewed intra-abdominal bleeding.

**Fig. 3:**
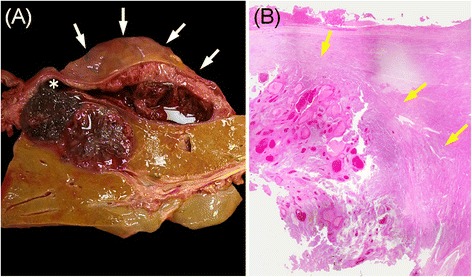
Cross-sectional and histologic findings of the liver at autopsy. The ruptured region (*white asterisk*) and subcapsular hematoma surrounding the liver (*white arrow*) (**a**). The metastatic tumor is well demarcated by a fibrous capsule (*yellow arrow*) (hematoxylin-eosin stain, ×40) (**b**)

**Fig. 4:**
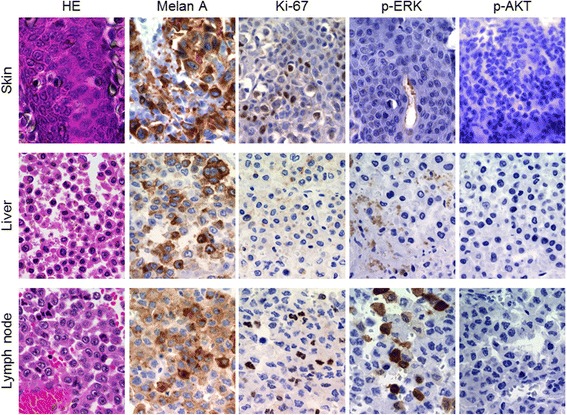
Microscopic findings of the skin, liver, and lymph node specimen. The skin lesion and the liver and lymph node metastases are stained to detect Melan-A, Ki-67, phosphorylated extracellular signal regulated kinase, and phosphorylated Akt (×400)

An autopsy examination revealed hemoperitoneum due to rupture of the liver metastases. Metastases were also discovered in the brain and lungs as well as in the kidneys, adrenal gland, and lymph nodes, although these had not been detected on imaging while she was alive. There was also massive bloody ascites (1700 mL). The background liver was completely normal, whereas exposed necrotic tissue and intratumoral hemorrhage were observed at the site of tumor rupture (Fig. 3). We concluded that the cause of death was hemorrhagic shock from ruptured hepatic metastases of malignant melanoma. Finally, for improved understanding of the mechanism of refractory metastasis, we conducted an immunohistochemical analysis of the signal transduction molecules, phosphorylated extracellular signal-regulated kinase (p-ERK), and phosphorylated Akt (p-Akt), as well as the melanocyte marker Melan-A and Ki-67 in tumor cells of the primary malignant melanoma obtained from the right lower leg and in hepatic and lymph node metastases obtained on autopsy (Fig. 4). Our findings showed that hepatic and lymph node metastases were positive for p-ERK and negative for p-AKT, even though the primary tumor was negative for both.

## Conclusions

Compared to primary hepatic tumors, hepatic metastases that result in spontaneous rupture are rare [6]. Furthermore, only eight reports describing rupture of hepatic metastases of malignant melanoma have been published [2–9].

The rupture of hepatic metastases is thought to be derived from tumor necrosis and increased intra-abdominal pressure due to straining upon defecation and forceful palpation [11–13]. In our autopsy case, gross examination and a microscopic study of the liver suggested that the increased intratumoral pressure by rapid growth, acute intratumoral bleeding, and the subsequent tumor necrosis resulted in rupture (Fig. 3).

The recently developed vemurafenib, a selective inhibitor of the mutant BRAF protein or gene product, has been reported to be extremely effective in patients with metastatic melanoma who harbor a *BRAF V600E* mutation [14–16]. Davies et al. reported that this mutation is present in approximately 50 % of cutaneous melanoma cases [16]. In our case, since the *BRAF* mutation was positive, we used vemurafenib for treatment, and the patient was responsive to it. In addition, Anker et al. described synergistic toxicity from the combination of radiation and vemurafenib [17], but fortunately, our patient did not experience complications, such as liver and skin toxicity, during the course of radiation and vemurafenib treatment. However, 2 months later, a part of the hepatic metastasis had enlarged and ruptured with an increased 5-S-CD.

The clinical course indicated that secondary resistance occurred during treatment with vemurafenib. As for the mechanism of secondary resistance to vemurafenib, tumor re-proliferation requires reactivation of the mitogen-activated protein kinase (MAPK) pathway (intrinsic pathway) or activation of the phosphatidylinositiol 3-kinase/AKT/mammalian target of rapamycin (PI3K/AKT/mTOR) pathway (extrinsic pathway), both of which have been reported [18, 19]. Therefore, we conducted an immunohistochemical analysis of the signal transduction molecules, p-ERK, and p-Akt, as well as the melanocyte marker Melan-A and Ki-67 (Fig. 4). Our findings showed that hepatic and lymph node metastases obtained on autopsy were positive for p-ERK and negative for p-AKT, even though the primary tumor was negative for both. These findings suggested the strong possibility that reactivation of the MAPK pathway had occurred without activation of the PI3K/AKT/mTOR pathway in the hepatic and lymph node metastases. This type of secondary resistance is thought to be derived from various causes such as *NRAS* or *MEK* mutations.

This case suggested that secondary resistance of vemurafenib, confirmed by an immunohistochemical study, may cause rapid tumor growth and subsequent rupture. In addition, immunohistochemical studies may clarify the mechanism underlying secondary resistance, and they may provide important information regarding the future treatment of vemurafenib-resistant metastatic melanoma.

## Consent

Written informed consent was obtained from the patient for publication of this Case report and any accompanying images. A copy of the written consent is available for review by the Editor of this journal.

## Abbreviations

TAE: Transcatheter arterial embolization
MAPK: Mitogen-activated protein kinase
DAV-feron: Doxorubicin, adriamycin, vincristine, and interferon beta
CT: Computed tomography
5-S-CD: 5-S-cysteinyldopa
PI3K/AKT/mTOR: Phosphatidylinositiol 3-kinase/AKT/mammalian target of rapamycin
p-ERK: Phosphorylated extracellular signal regulated kinase
p-Akt: Phosphorylated Akt
